# Long Non-Coding RNA LINC01569 Promotes Proliferation and Metastasis in Colorectal Cancer by miR-381-3p/RAP2A Axis

**DOI:** 10.3389/fonc.2021.727698

**Published:** 2021-08-06

**Authors:** Guang-yao Ye, Zi-zhen Zhang, Chun-chao Zhu, Zhi-jie Cong, Zhe Cui, Lu Chen, Gang Zhao

**Affiliations:** Department of Gastrointestinal Surgery, Ren Ji Hospital, School of Medicine, Shanghai Jiao Tong University, Shanghai, China

**Keywords:** long non-coding RNA (lncRNA) LINC01569, miR-381-3p, RAP2A, colorectal cancer, metastasis, biomarker

## Abstract

**Background:**

Long non-coding RNAs (lncRNAs) display regulatory function flexibly in tumor onset and developments. Our study aimed to delve into the roles of lncRNA LINC01569 (LINC01569) in colorectal cancer (CRC) progression to study the potential mechanisms.

**Methods:**

The genetic expression profiles of miR-381-3p and LINC01569 were measured by RT-PCR. The subcellular localization of LINC01569 in CRC cells was identified using subcellular fractionation location. Loss-of-function assays were performed to explore the potential effects of LINC01569 on CRC progression. Dual-luciferase reporter analysis was employed to verify the binding connections among LINC01569, miR-381-3p, and RAP2A.

**Results:**

LINC01569 expression was distinctly increased in CRC. Curiously, if LINC01569 is removed, CRC cells will not migrate, proliferate, and invade remarkably. Molecular mechanism exploration uncovered that LINC01569 acted as a ceRNA competing with RAP2A to bind with miR-381-3p. Furthermore, rescue experiments corroborated the fact that miR-381-3p suppression reversed the inhibitory actions of LINC01569 knockdown on the expression of RAP2A and CRC progression.

**Conclusion:**

Overall, our findings indicate that LINC01569 plays a key role in CRC development by means of aiming at the miR-381-3p/RAP2A axis and can be equivalent to an underlying medicinal target to save CRC patients.

## Introduction

Colorectal cancer (CRC) is one of the most common types of malignancy and the third leading cause of cancer-associated mortality worldwide (1). More than 2,200,000 new CRC patients and 1,100,000 CRC-associated mortality will be seen each year by 2020 (2, 3). The CRC prognosis remains dismal even if recent remarkable developments of intervention and diagnosis in the early stage have been achieved (4, 5). More than half of the advanced-stage patients pass away due to metastasis and recurrence (6, 7). Hence, there is an urgent need to find useful biomarkers that may contribute to disease management for CRC patients.

Long non-coding RNAs (lncRNAs) belong to a type of RNAs without coding capacity (8). In mammals, lncRNAs can function as important regulators in alternative splicing of endogenous pre-mRNAs, chromatin state control, transcriptional and post-transcriptional gene regulation (9, 10). Participating in various diseases, especially cancer, by abundant proof confirmation, lncRNAs cast impacts on certain processes biologically and pathologically, including metabolism, drug resistance, metastasis, and cell proliferation (11–13). For instance, lncRNA LINC00313 is shown to exhibit an increased level in CRC and promote cancer metastasis and proliferation by regulating miR-4677-3p/CDK6 axis (14). Therefore, the functional characterization of critical lncRNAs in cancer biology provides novel insights into the development of novel cancer biomarkers and targeted therapies.

LncRNA LINC01569 (LINC01569), located at 16p13.3, is a novel lncRNA whose function in vital movement remains largely unclear. Previously, LINC01569 was shown to mediate glucocorticoid effects on mechanotransduction by destabilizing messenger RNA (mRNA) of mechanosensors (15). However, whether LINC01569 is involved in tumor progression has not been reported. We firstly delivered proofs to show that the expression of LINC01569 expression was remarkably increased in patients with CRC in this research. Then, we performed a series of functional assays to explore its effects on CRC progression. Our findings suggested LINC01569 as a potential target in the CRC diagnosis and therapy.

## Materials and Methods

### Human Samples

Twelve CRC tissues in pairs and tissues in normal status nearby stored within liquid nitrogen were collected from Ren Ji Hospital, School of Medicine, Shanghai Jiao Tong University from June 2019 to June 2020. All clinical samples were confirmed by at least two pathologists. The characteristics of 12 CRC patients were shown in **Table 1**. All the patients signed informed consent, and the ethical committee of Ren Ji Hospital, School of Medicine, Shanghai Jiao Tong University approved this study (Permit number:RJH-0139). Under the guidance of the Declaration of Helsinki, we conducted all clinical experiments.

**Table 1:** Clinicopathological characteristics of 12 CRC patients.

Characteristics	No. of patients
Age	
<55	5
≥55	7
Gender	
Male	8
Female	4
Tumor size	
<4 cm	7
≥4 cm	5
Histology/differentiation	
Well + Moderate	7
Poor	5
TNM stage	
I, II	8
III	4
Distant metastasis	
Present	2
Absent	10

### Cell Culture

We purchased the CRC cell lines (SW620, HCT116, SW480 and HT29) along with the colonic epithelial cell line NCM460 from National Biomedical Laboratory (Haidian, China). In medium RPMI-1640 (PM150110, Procell, Wuhan, China) supplemented with 10% FBS (GIBCO, Guangzhou, China), 100 U/ml penicillin and 100 mg/ml streptomycin), cells were cultivated. Cells were kept within the incubator at 5% CO_2_ with a temperature of 37°C.

### Cell Transfection

Genomics substances were provided by Liaoning Biology (Wuhan, Hubei, China), including negative control (NC) plasmids, miR-381-3p inhibitors, miR-381-3p mimics, and short hairpin RNA for LINC01569 (sh-LINC01569-1), and LINC01569 (sh-LINC01569-2). The transfections of all substances into SW620 and HCT116 cells were carried out for 48 h using Lipofectamine 2000 Reagent (ThermoFisher,Shenzhen, Guandong, China).

### Real-Time Quantitative PCR

Following the standard process, TRIzol reagent (HaiGene, Haerbing, China) was applied for the extraction of total RNAs from clinical samples and human specimens. Following the instructions of the manufacturer, total RNAs were transcribed reversely into cDNA by utilizing a PrimeScript™ RT reagent kit (HaiGene, Haerbing, China). RT-PCR was performed by applying ABI 7900HT Fast Real-Time PCR System. GAPDH or U6 served as the endogenous control, and relative expression was compared by the 2^-ΔΔCt^ method. Primer sequences were shown in **Table 2**.

**Table 2:** The primers used in this study for RT-PCR.

Names	Sequences (5′–3′)
LINC01569: Forward	CAGTGCCACCTTCTCTACCTGCT
LINC01569: Reverse	GGCATGACCTCACACTCACGC
miR-381-3p: Forward	TCTGTCTCACTAATTGCTCTCCT
miR-381-3p: Reverse	TATGGTTGTTCTGCTCTCTGTCTC
RAP2A: Forward	ATGCGCGAGTACAAAGTGGT
RAP2A: Reverse	GCGACGAATCCACCTCGAT
GAPDH: Forward	ACCACAGTCCATGCCATCAC
GAPDH: Reverse	TCCACCACCCTGTTGCTGTA
U6: Forward	GCGCGTCGTGAAGCGTTC
U6: Reverse	GTGCAGGGTCCGAGGT

### Cell Proliferation

For CCK-8 assays, cells were plated into plates with 96 wells and cultivated according to the indicated days. Subsequently, CCK-8 solution was added and incubated for 2 h. An absorbance of 450 nm was eventually recorded by utilizing a microplate reader. For colony formation assays, cells were plated into plates with 6 wells and cultured for 14 days. Subsequently, methanol was used to fix clones, followed by dyeing with 0.1% crystal violet. Finally, our group calculated the colony numbers.

### EdU Staining Assay

By using EdU (5-ethynyl-20-deoxyuridine) analysis resulting from Cell-Light EdU DNA Cell Proliferation Kit, we surveyed the proliferation of cells. Cultured at 5% CO_2_ with a temperature of 37°C for 48 h, the 1 × 10^4^ cells were seeded into plates with 96 wells. Every well with cells cultivated for as long as 2 h was added with Edu solution with a 50 μM of eventual concentration. After that, cells were fixed by using 4% paraformaldehyde and dyed by applying Apollo dye solution for proliferation exploration. By using a fluorescence microscope, the observation, calculation, and photographing of the cells with positive EdU were carried out after we washed the cells with PBS (Olympus, Tokyo, Japan).

### Wound-Healing Assays

The SW620 and HCT116 cells (6 × 10^5^ density) were seeded into plates with 12 wells and grown until reaching 80% confluence. Subsequently, the linear scratch of the cell monolayer was carried out by using the tip of a sterile pipette. At 0 and 48 h post-wounding, 5% paraformaldehyde was used to fix the cells for a quarter followed by image recordings for the calculation of the migratory cells.

### Transwell Assays

For the detection of cellular invasion, a transwell chamber (Quasi Vivo, Beijing, China) was used. After the upper chamber was coated with Matrigel, cells were plated onto them. The chamber at the bottom was provided with the complete medium which carried 10% FBS (Gibico, USA). After 24 h, the lower chamber was provided with crystal violet at a concentration of 0.1 and 20% methanol. Under the microscope, we calculated the cells of invasion.

### Subcellular Fractionation Location

To separate the RNAs from nuclear or cytoplasm fraction, our group used a Nuclear/Cytosol Fractionation Kit (Biovision, Shenzhen, Guangdong, China). The levels of LINC01569 in cytoplasm or nuclear components were examined using subcellular fractionation location assays (16). GAPDH and U6 separately served as nuclear or cytoplasm controls.

### Animal Experiments

Abiding by the accord accepted by the Animal Care and Use Committee of Ren Ji Hospital, School of Medicine, Shanghai Jiao Tong University, animal experiments were conducted to explore the function of LINC01569 knockdown. Briefly, 6 × 105 cells with sh-LINC01569 or sh-NC stable transfection were injected into the flank of nude mice (n = 6 per group). Every 7 days, our group measured the width and length of tumors. According to the formula (length × (width)^2^/2), volume was calculated. All mice were grown for 28 days, followed by sacrifice for the collection of tumor specimens.

### Luciferase Reporter Assays

Enlarged *via* the use of PCR, the mutant types (MUT) along with the wild ones (WT) of RAP2A, which carried the assumed binding area to couple with miR-381-3p, were duplicated into the reporter vector of pmirGLO luciferase (Promega, China). Then, cells were co-transfected with miR-381-3p mimics or miR-NC and luciferase vector containing 3′-UTR of RAP2A, wild-type (Wt) or mutant (Mut) RAP2A fragment, using Lipofectamine 2000. Luciferase reporter assay was performed using the Dual-Luciferase Reporter Assay System (Promega) 48 h later.

### Western Blot

Through the application of RIPA lysis buffer which carried proteinase restrainer, we acquired and lysed SW620 and HCT116 cell lines (Roche, China). By applying the BCA protein analysis tool, we quantitated the overall protein (Pierce, China). Before incubating with secondary antibodies linked to HRP, proteins blotted onto PVDF membrane were severed *via* SDS-PAGE at a density of 10%, then cultivated overnight along with primary antibodies at a temperature of 4°C to go against PAP2A. The primary antibodies and second antibodies were purchased from CWBIO (Beijing, China). Finally, ECL chemiluminescence kit (Beyotime) was used to visualize blots.

### Statistical Analysis

All statistical analyses were performed using SPSS 18.0 software (IBM) and Prism 7 (GraphPad Software, USA). All assays were conducted in triplicate. Differences between two independent groups were tested with the Student’s t-test. The one-way ANOVA was used to analyze three or more groups. A p-value <0.05 was considered statistically significant.

## Results

### LINC01569 Levels Were Upregulated in CRC

**Figure 1:**
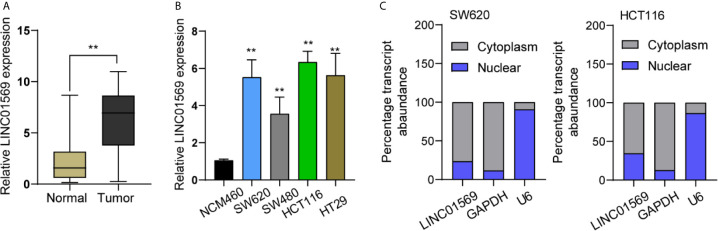
LINC01569 expression was upregulated in CRC and was correlated with prognosis. **(A)** qRT-PCR assays of the expression of LINC01569 in a 12-pair CRC specimen and corresponding non-tumor samples. **(B)** The expression of LINC01569 within CRC cell lines (SW620, HCT116, SW480, and HT29) and the epithelial colonic cell line (NCM460) *via* qRT-PCR analysis. **(C)** Relative LINC01569 amount within nuclear along with cytosolic fractions of SW620 and HCT116 cells. **P < 0.01.

Primarily, whether LINC01569 levels were dysregulated in CRC was examined by using RT-PCR. **Figure 1A** shows that the expression of LINC01569 in specimens with CRC was distinctly stronger than that in the matched specimens without tumors. In cellular experiments by RT-PCR, our research discovered that LINC01569 expression was distinctly enhanced in four CRC cell lines compared with NCM460 cells (**Figure 1B**). Subcellular fractionation location assays showed that LINC01569 was mainly expressed in the cytoplasm of SW620 and HCT116 cells (**Figure 1C**).

### Knockdown of LINC01569 Suppressed the Proliferation and Metastasis of CRC Cells

**Figure 2:**
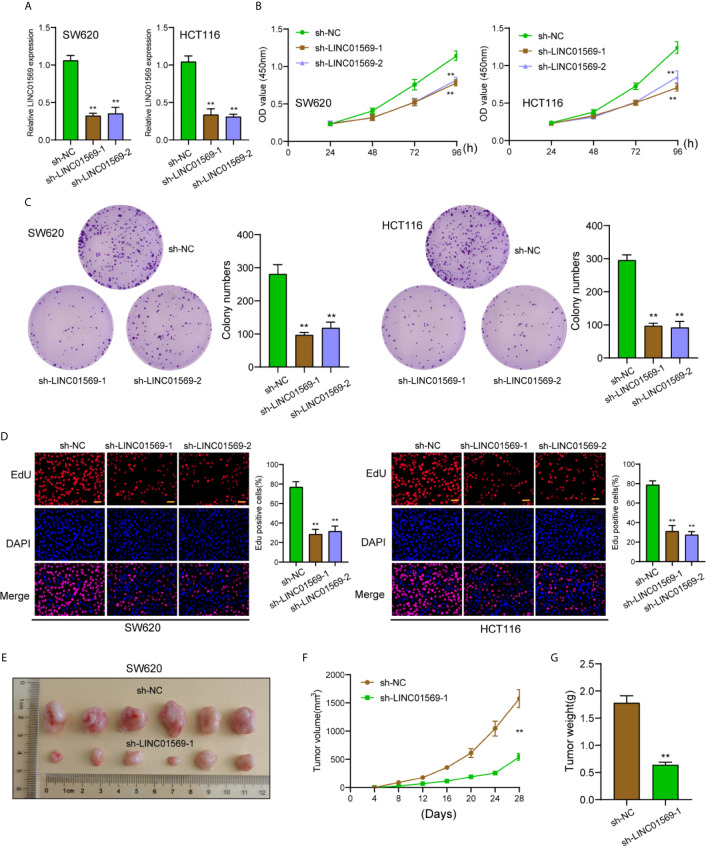
LINC01569 promoted the proliferation of CRC cells. **(A)** qRT-PCR assay for LINC01569 expression in SW620 and HCT116 cells transfected with sh-LINC01569-1, sh-LINC01569-2, or sh-NC cells at 48 h post-transfection. **(B, C)** Cell viability of LINC01569-silenced CRC cells was measured by CCK-8 assay and colony-generating assay. **(D)** EdU assay for the functional exploration of LINC01569 knockdown in CRC cells. **(E)** Representative photographs of tumor xenografts obtained from the sh-LINC01569-1 as well as sh-NC groups. **(F)** The growth curves for the tumor xenografts. **(G)** The weight of the tumor xenografts. **P < 0.01.

**Figure 3:**
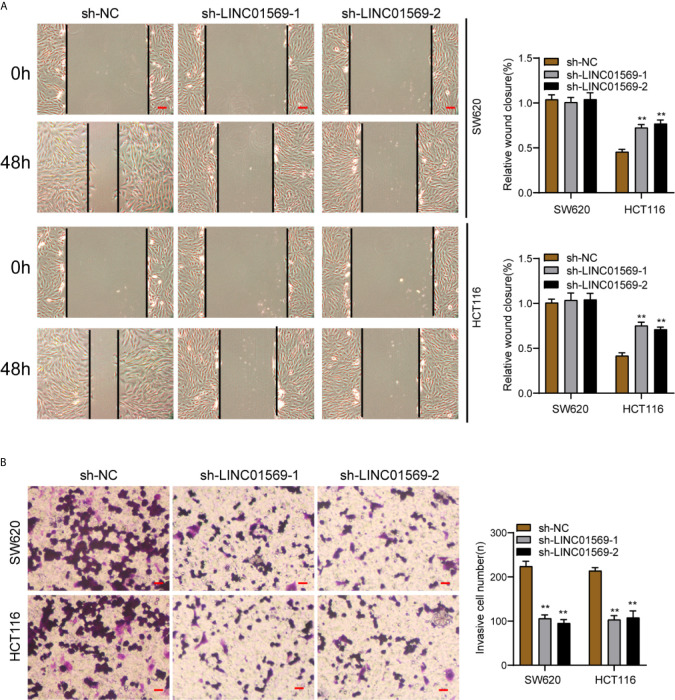
LINC01569 promoted the migration and invasion of CRC cells. **(A)** Wound healing analysis of migratory rate in SW620 and HCT116 cells transfected with sh-NC or sh-LINC01569-1. **(B)** Transwell analysis of invasion in transfected SW620 and HCT116 cells. **P < 0.01.

The biological function of LINC01569 in SW620 and HCT116 cells was assessed *via* loss-of-function methods. The interference potency of two shRNAs was displayed in **Figure 2A**, which indicated that sh-LINC01569-1 and sh-LINC01569-2 efficiently reduced LINC01569 expression and were applied in the assays afterwards. CCK-8 and colony-generating assays suggested that knockdown of LINC01569 impaired CRC cell proliferation (**Figures 2B, C**). EdU assays also revealed that the knockdown of LINC01569 led to a decreased growth of cells (**Figure 2D**). To further examine the function of LINC01569 in CRC, we performed *in vivo* assays. The volume and weight of tumors in the LINC01569 (LINC01569 knockdown) group were significantly smaller than that in the control group (**Figures 2E–G**). Subsequently, we carried out wound-healing assays and transwell migration to assess the impacts of LINC01569 on the metastatic ability of CRC cells. The ability to migrate and invade for both SW620 and HCT116 cells was distinctly inhibited with the depletion of LINC01569 as predicted (**Figures 3A, B**).

### LINC01569 Directly Interacts With miR-381-3p

**Figure 4:**
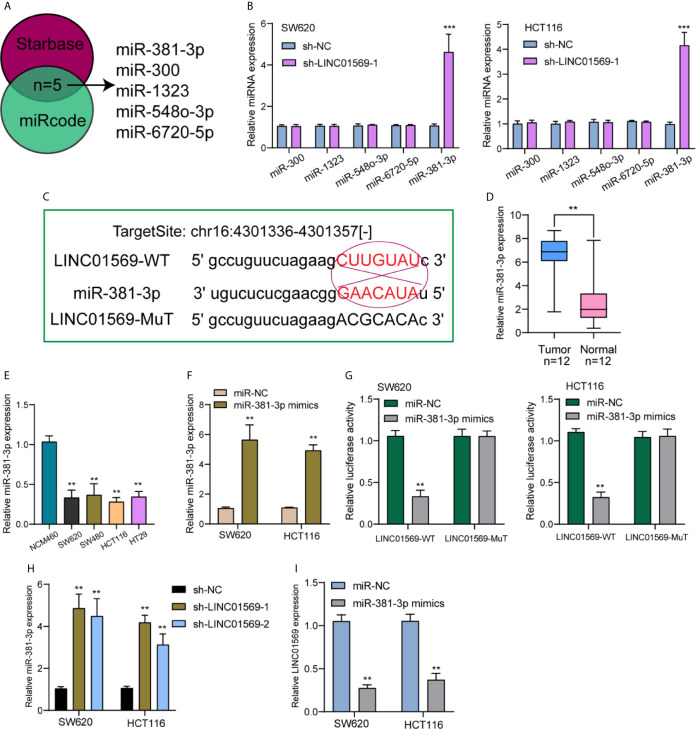
MiR-381-3p served as a straight target of LINC01569. **(A)** Potential miRNAs that sponged LINC01569 were predicted using the Starbase 2.0 and miRcode. **(B)** The expression of the five miRNAs in SW620 and HCT116 cells transfected with sh-NC or sh-LINC01569-1. **(C)** Starbase database projected an underlying adhesion area for LINC01569 together with miR-381-3p. **(D)** RT-PCR for the expression of LINC01569 in our patients. **(E)** miR-381-3p levels were decreased in four CRC cell lines. **(F)** The transfection efficiency of miR-381-3p mimics was demonstrated in SW620 and HCT116 cells using RT-PCR. **(G)** The mutual effects of miR-381-3p and LINC01569 were displayed by luciferase reporter analysis. **(H)** The distinct downregulation of miR-381-3p was confirmed in SW62 and HCT116 cells transfected with sh-LINC01569-1 or sh-LINC01569-2. **(I)** miR-381-3p mimics inhibited the LINC01569 expression in SW62 and HCT116 cells. **P < 0.01, ***P < 0.001.

Previously, we have confirmed the location of a majority of LINC01569 in the cytoplasm of SW620 and HCT116 cells, and it has been demonstrated that numerous cytoplasmic lncRNAs play a role of competing endogenous RNAs (ceRNAs) *via* competitively binding microRNAs. Using Venn, five targeting miRNAs (miR-381-3p, miR-300, miR-1323, miR-548-3p, and miR-6720-5p) of LINC01569 were screened from Starbase 2.0 and miRCode database (**Figure 4A**). After knockdown of LINC01569, only the expression of miR-381-3p was distinctly increased in both SW620 and HCT116 cells (**Figure 4B**). Thus, we chose miR-381-3p as the research target. The target site was chr16:4301336–4301357[−], and the combined sequences were shown in **Figure 4C**. RT-PCR showed miR-381-3p expression was remarkably increased in both samples and cell lines of CRC, and its diagnostic significance was also demonstrated with an AUC = 0.8062 (**Figures 4D, E**). Within SW620 and HCT116 cells, its levels were upregulated after the transfection of imitators of miR-381-3p (**Figure 4F**). The outcome of the luciferase reporter analysis revealed that before the 1/2 decrease of the density of fluorescence, LINC01569 wild-type was jointly transfected with miR-381-3p imitators; nevertheless, as to SW620 as well as HCT116 cell lines, other groups were identical (**Figure 4G**). Finally, RT-PCR assays confirmed that the depletion of LINC01569 remarkably facilitated the miR-381-3p expression, while miR-381-3p overexpression exhibited an opposite effect (**Figures 4H, I**).

### RAP2A Was a Downstream Target Gene of miR-381-3p

**Figure 5:**
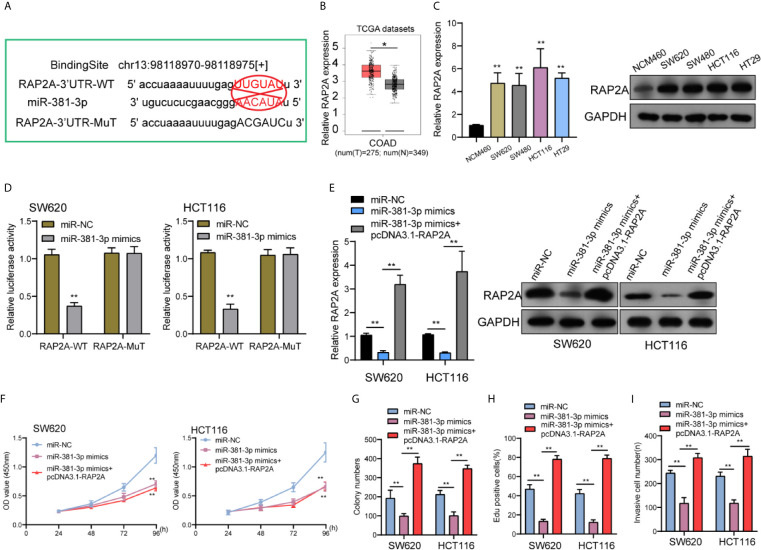
miR-381-3p directly interacted with RAP2A in CRC. **(A)** Schematic outlining the predicted binding sites between miR-381-3p and RAP2A. **(B)** A larger amount of RAP2A was observed in CRC specimens compared with non-tumor specimens from TCGA datasets. **(C)** RT-PCR and Western blot examined the levels of RAP2A in four CRC cells and NCM460 cells. **(D)** Luciferase reporter assays for the determination of the interacting activity between miR-381-3p and RAP2A. **(E)** The expression of RAP2A in SW620 and HCT116 cells transfected with miR-NC; miR-381-3p mimics or miR-381-3p mimics + RAP2A. **(F–I)** The functional assays including **(F)** CCK-8, **(G)** colony numbers, **(H)** Edu assays, and **(I)** transwell assays in W620 and HCT116 cells transfected with miR-NC, miR-381-3p mimics or miR-381-3p mimics + RAP2A. **P < 0.01, *P < 0.05.

To find the mRNAs that were involved in LINC01569/miR-381-3p regulation of CRC activity, Starbase 2.0 was used to find the possible genes. We paid attention to RAP2A which had been demonstrated to play a role of a tumor promoter for several tumors, including CRC (**Figure 5A**) (17, 18). A distinct upregulation of RAP2A was also observed in CRC samples in contrast to normal rectum samples from TCGA datasets (**Figure 5B**). RT-PCR and Western blot also displayed an upregulated level of RAP2A in four CRC cell lines in contrast to NCM460 cells (**Figure 5C**). Luciferase reporter assays showed that in contrast to the other group of vector, in SW620 and HCT116 cells, the joint transfection of miR-381-3p with Wt-3′UTR remarkably inhibited the activities of luciferase, whereas the luciferase activities of Mut-3′UTR were stable (**Figure 5D**). Further experiments indicated that the transfection of pcDNA3.1-RAP2A reversed the distinct suppression of miR-381-3p overexpression on the expression of RAP2A (**Figure 5E**). In addition, a series of functional assays confirmed that miR-381-3p overexpression suppressed the ability to proliferate for SW620 as well as HCT116 cells, while the overexpression of RAP2A reversed the above suppression (**Figures 5F–H**). In addition, a similar result was observed in invasion ability (**Figure 5I**).

### LINC01569 Promoted Tumor Progression of CRC Cells by Increasing RAP2A Expression *via* Sponging miR-381-3p

**Figure 6:**
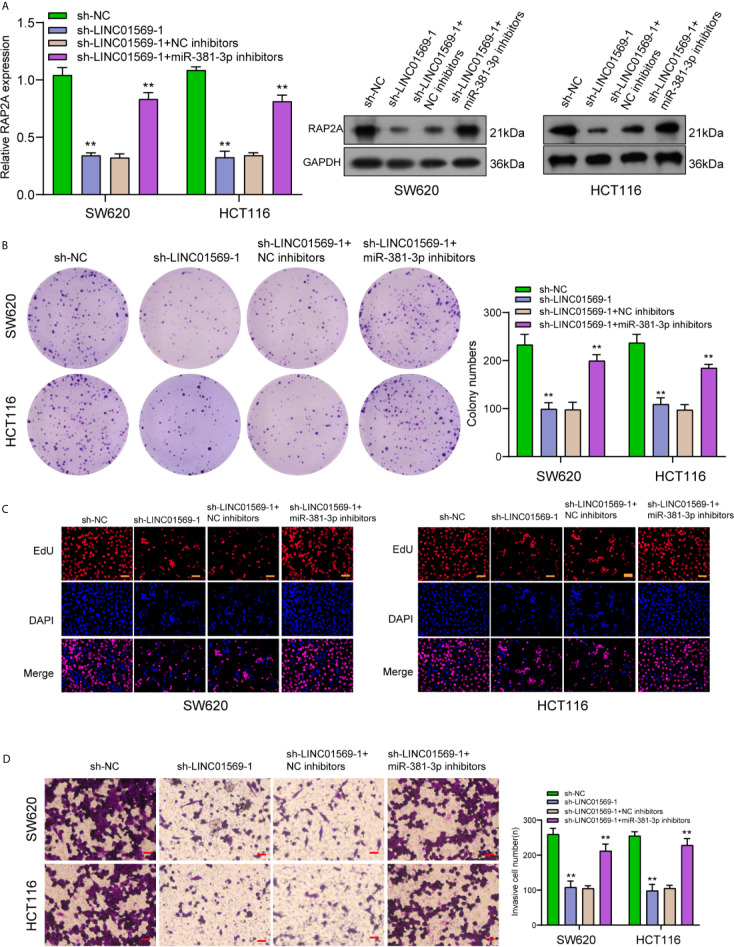
Downregulation of miR-381-3p rescued LINC01569-induced changes in CRC cell lines. **(A)** RT-PCR and Western blot determined the expression of RAP2A in SW620 and HCT116 cells transfected with sh-NC, sh-LINC01569-1, sh-LINC01569-1 + NC inhibitors or sh-LINC01569-1 + miR-381-3p inhibitors. **(B–D)** A series of functional assays in SW620 and HCT116 cells transfected with sh-NC, sh-LINC01569-1, sh-LINC01569-1 + NC inhibitors or sh-LINC01569-1 + miR-381-3p inhibitors. **P < 0.01.

To further explore the biological interactions among LINC01569, miR-381-3p, and RAP2A in CRC, SW620 and HCT116 cells were co-transfected with sh-LINC01569 and miR-381-3p inhibitor. As presented in **Figure 6A**, we observed that the silence of miR-381-3p distinctly reversed the suppression of LINC01569 knockdown on the expression of RAP2A. Then, by the use of a series of functional assays, our group found that the ability of proliferation and invasion was suppressed in sh-LINC01569-1 + NC inhibitor group and recovered in sh-LINC01569-1 + miR-381-3p inhibitor group (**Figures 6B–D**).

## Discussion

Up till the present moment, on account of metastasis and recurrence, the incidence and death rates of CRC remain high (19). At the same time, reliable and specific biomarkers to prognose and diagnose CRC are scarce and lack exploration. In recent years, increasing research studies have reported the potential of lncRNAs used as novel biomarkers of prognosis and diagnosis for tumor patients, including CRC patients (20, 21). In this study, our team recognized a new type of lncRNA regarding CRC, LINC01569 which was highly expressed in CRC specimens and cell lines. In order to unveil the possible role of LINC01569 within CRC development, a number of function-related assays were carried out, and we observed that knockdown of LINC01569 suppressed the proliferation, migration, and invasion of SW620 and HCT116 cells. Previously, several lncRNAs have been reported to play a role of cancer suppressors or oncogenes for CRC progression in both *in vivo* and *in vitro* assays (22, 23). In addition, the tumor-related function of lncRNAs in different types of tumors may not be consistent. We searched Google scholar and Pubmed and did not found the functional assays of LINC01569 in any types of tumors. Results from this research may deliver a CRC therapeutic target new to the world and may also show a new clue for the biological study of LINC01569 in other tumors.

Accumulating evidence indicated that lncRNAs are used as ceRNAs of endogenous miRNAs to bind to miRNAs, and this function competitively inhibited the binding of miRNAs to their targets (24–26). For instance, lncRNA MCM3AP-AS1 facilitated the ability to invade and proliferate for CRC cells through sponging miR-193a-5p/SENP1 (27). LncRNA LINC00511 exhibited oncogenic roles *via* decreasing NFIA through sponging miR-29c-3p (28). Thus, we wondered whether LINC01569 may also show its effects *via* a similar mechanism. Subcellular fractionation location confirmed the location of LINC01569 in the cytoplasm, which supported the probability of LINC01569 acting as a ceRNA. Thus, we performed a series of experiments, confirming that LINC01569 acted as ceRNA for miR-381-3p and inhibited its functioning. Previously, in accord with our results, miR-381-3p registered low expression in some cancers such as CRC and suppressed the proliferation and metastasis of cancer cells (29, 30). Thus, there is a possibility that LINC01569 serves as a carcinogen through sponging miR-381-3p.

Related to various cell activities such as the ability to proliferate, bind, and migrate, Rap-2A (RAP2A), a protein regarding Ras, presented as one of the massive family of small GTPase protein and a p53 transcription target (31, 32). Moreover, *via* initiating the signaling pathway of AKT, the ability to invade, migrate, and metastasize for tumor cells was facilitated by RAP2A (33). In CRC, RAP2A displayed high expression, serving as a cancer promotor (17). In addition, miR-381-3p may target RAP2A to suppress its expression. Our research also provided evidence that there was an overexpression of miR-381-3p in CRC cells, followed by Dual-luciferase reporter assays and rescue experiments confirming the targeting combination for miR-381-3p along with RAP2A, which was consistent with previous findings. Moreover, primarily, our research delivered proof that LINC01569 facilitated the capability to proliferate and metastasize for CRC cells by increasing RAP2A through sponging miR-381-3p. Thus, our findings may provide a novel clue for the targeted therapy *via* targeting LINC01569/miR-381-3p/RAP2A axis.

We acknowledged some limitations in this study. First, the sample size was relatively small; we will collect more samples to explore the clinical significance of LINC01569 in CRC patients in future studies. Second, we did not explore the potential mechanisms involved in LINC01569 overexpression in CRC.

## Conclusions

Our study identified a novel lncRNA LINC01569 associated with poor prognosis in CRC. Upregulation of LINC01569 facilitated the capability to proliferate and metastasize for CRC cells by targeting miR-381-3p/RAP2A. Therefore, the targeted regulation of the LINC01569/miR-381-3p/RAP2A axis improved the condition of the disease.

## Data Availability Statement

The raw data supporting the conclusions of this article will be made available by the authors, without undue reservation.

## Ethics Statement

The studies involving human participants were reviewed and approved by Ren Ji Hospital, School of Medicine, Shanghai Jiao Tong University. The patients/participants provided their written informed consent to participate in this study. The animal study was reviewed and approved by Ren Ji Hospital, School of Medicine, Shanghai Jiao Tong University.

## Author Contributions

G-yY and Z-zZ wrote the main manuscript and analyzed the data. C-cZ, G-yY, Z-jC, ZC, and LC performed the experiments. G-yY, Z-zZ, and GZ designed the study. All authors contributed to the article and approved the submitted version.

## Conflict of Interest

The authors declare that the research was conducted in the absence of any commercial or financial relationships that could be construed as a potential conflict of interest.

## Publisher’s Note

All claims expressed in this article are solely those of the authors and do not necessarily represent those of their affiliated organizations, or those of the publisher, the editors and the reviewers. Any product that may be evaluated in this article, or claim that may be made by its manufacturer, is not guaranteed or endorsed by the publisher.

## References

[1] Dekker E Tanis PJ Vleugels JLA Kasi PM Wallace MB. Colorectal Cancer. Lancet (2019) 394:1467–80. 10.1016/S0140-6736(19)32319-0 31631858

[2] Wong SH Yu J. Gut Microbiota in Colorectal Cancer: Mechanisms of Action and Clinical Applications. Nat Rev Gastroenterol Hepatol (2019) 16:690–704. 10.1038/s41575-019-0209-8 31554963

[3] Siegel RL Miller KD Goding Sauer A Fedewa SA Butterly LF Anderson JC. Colorectal Cancer Statistics, 2020. CA Cancer J Clin (2020) 70:145–64. 10.3322/caac.21601 32133645

[4] Ocvirk S O’Keefe SJD. Dietary Fat, Bile Acid Metabolism and Colorectal Cancer. Semin Cancer Biol (2021) 73:347–55. 10.1016/j.semcancer.2020.10.003 33069873

[5] Si H Yang Q Hu H Ding C Wang H Lin X. Colorectal Cancer Occurrence and Treatment Based on Changes in Intestinal Flora. Semin Cancer Biol (2021) 70:3–10. 10.1016/j.semcancer.2020.05.004 32404293

[6] Piawah S Venook AP. Targeted Therapy for Colorectal Cancer Metastases: A Review of Current Methods of Molecularly Targeted Therapy and the Use of Tumor Biomarkers in the Treatment of Metastatic Colorectal Cancer. Cancer (2019) 125:4139–47. 10.1002/cncr.32163 31433498

[7] Lech G Słotwiński R Słodkowski M Krasnodębski IW. Colorectal Cancer Tumour Markers and Biomarkers: Recent Therapeutic Advances. World J Gastroenterol (2016) 22:1745–55. 10.3748/wjg.v22.i5.1745 PMC4724606 26855534

[8] Kopp F Mendell JT. Functional Classification and Experimental Dissection of Long Noncoding RNAs. Cell (2018) 172:393–407. 10.1016/j.cell.2018.01.011 29373828 PMC5978744

[9] Ali T Grote P. Beyond the RNA-Dependent Function of LncRNA Genes. Elife (2020) 9:1–14. 10.7554/eLife.60583 PMC7584451 33095159

[10] McCabe EM Rasmussen TP. lncRNA Involvement in Cancer Stem Cell Function and Epithelial-Mesenchymal Transitions. Semin Cancer Biol (2020). 10.1016/j.semcancer.2020.12.012 33346133

[11] Goodall GJ Wickramasinghe VO. RNA in Cancer. Nat Rev Cancer (2021) 21:22–36. 10.1038/s41568-020-00306-0 33082563

[12] Gil N Ulitsky I. Regulation of Gene Expression by Cis-Acting Long Non-Coding RNAs. Nat Rev Genet (2020) 21:102–17. 10.1038/s41576-019-0184-5 31729473

[13] Kim T Croce CM. MicroRNA and ER Stress in Cancer. Semin Cancer Biol (2021). 10.1016/j.semcancer.2020.12.025 33422566

[14] Zhai Y Liu Y Wang Z Wang W Zhou J Lu J. Long Non-Coding RNA LINC00313 Accelerates Cervical Carcinoma Progression by miR-4677-3p/CDK6 Axis. Onco Targets Ther (2021) 14:2213–26. 10.2147/OTT.S265007 PMC8018412 33824592

[15] Zhu H Li J Li Y Zheng Z Guan H Wang H. Glucocorticoid Counteracts Cellular Mechanoresponses by LINC01569-Dependent Glucocorticoid Receptor-Mediated mRNA Decay. Sci Adv (2021) 7:1–18. 10.1126/sciadv.abd9923 PMC7904261 33627425

[16] Wu Y Hu L Liang Y Li J Wang K Chen X. Up-Regulation of lncRNA CASC9 Promotes Esophageal Squamous Cell Carcinoma Growth by Negatively Regulating PDCD4 Expression Through EZH2. Mol Cancer (2017) 16:150. 10.1186/s12943-017-0715-7 28854977 PMC5577767

[17] Han Y Wang X Mao E Shen B Huang L. lncRNA FLVCR1−AS1 Drives Colorectal Cancer Progression *via* Modulation of the Mir−381/RAP2A Axis. Mol Med Rep (2021) 23:1–10. 10.3892/mmr.2020.11778 PMC7751490 33313944

[18] Zhang J Wei Y Min J Wang Y Yin L Cao G. Knockdown of RAP2A Gene Expression Suppresses Cisplatin Resistance in Gastric Cancer Cells. Oncol Lett (2020) 19:350–8. 10.3892/ol.2019.11086 PMC6923840 31897147

[19] Keller DS Windsor A Cohen R Chand M. Colorectal Cancer in Inflammatory Bowel Disease: Review of the Evidence. Tech Coloproctol (2019) 23:3–13. 10.1007/s10151-019-1926-2 30701345

[20] Chan JJ Tay Y. Noncoding RNA : RNA Regulatory Networks in Cancer. Int J Mol Sci (2018) 19:1–26. 10.3390/ijms19051310 PMC5983611 29702599

[21] Huarte M. The Emerging Role of lncRNAs in Cancer. Nat Med (2015) 21:1253–61. 10.1038/nm.3981 26540387

[22] Wang Y Lu JH Wu QN Jin Y Wang DS Chen YX. LncRNA LINRIS Stabilizes IGF2BP2 and Promotes the Aerobic Glycolysis in Colorectal Cancer. Mol Cancer (2019) 18:174. 10.1186/s12943-019-1105-0 31791342 PMC6886219

[23] Liang ZX Liu HS Wang FW Xiong L Zhou C Hu T. LncRNA RPPH1 Promotes Colorectal Cancer Metastasis by Interacting With TUBB3 and by Promoting Exosomes-Mediated Macrophage M2 Polarization. Cell Death Dis (2019) 10:829. 10.1038/s41419-019-2077-0 31685807 PMC6828701

[24] Tay Y Rinn J Pandolfi PP. The Multilayered Complexity of ceRNA Crosstalk and Competition. Nature (2014) 505:344–52. 10.1038/nature12986 PMC4113481 24429633

[25] Karreth FA Pandolfi PP. ceRNA Cross-Talk in Cancer: When Ce-Bling Rivalries Go Awry. Cancer Discovery (2013) 3:1113–21. 10.1158/2159-8290.CD-13-0202 PMC3801300 24072616

[26] Xiao C Nemazee D Gonzalez-Martin A. MicroRNA Control of B Cell Tolerance, Autoimmunity and Cancer. Semin Cancer Biol (2020) 64:102–7. 10.1016/j.semcancer.2019.04.004 PMC7295097 32522353

[27] Wang Y Yang L Chen T Liu X Guo Y Zhu Q. A Novel lncRNA MCM3AP-AS1 Promotes the Growth of Hepatocellular Carcinoma by Targeting miR-194-5p/FOXA1 Axis. Mol Cancer (2019) 18:28. 10.1186/s12943-019-0957-7 30782188 PMC6381672

[28] Hu Y Zhang Y Ding M Xu R. LncRNA LINC00511 Acts as an Oncogene in Colorectal Cancer *via* Sponging miR-29c-3p to Upregulate NFIA. Onco Targets Ther (2020) 13:13413–24. 10.2147/OTT.S250377 PMC7847767 33536761

[29] Zhang W Li X Zhang W Lu Y Lin W Yang L. The LncRNA CASC11 Promotes Colorectal Cancer Cell Proliferation and Migration by Adsorbing miR-646 and miR-381-3p to Upregulate Their Target Rab11fip2. Front Oncol (2021) 11:657650. 10.3389/fonc.2021.657650 33937069 PMC8084185

[30] Yang X Ruan H Hu X Cao A Song L. miR-381-3p Suppresses the Proliferation of Oral Squamous Cell Carcinoma Cells by Directly Targeting FGFR2. Am J Cancer Res (2017) 7:913–22. PMC5411798 28469963

[31] Minato N. Rap G Protein Signal in Normal and Disordered Lymphohematopoiesis. Exp Cell Res (2013) 319:2323–8. 10.1016/j.yexcr.2013.04.009 23603280

[32] Huang H Di J Qu D Gao Z Zhang Y Zheng J. Role of Rap2 and Its Downstream Effectors in Tumorigenesis. Anticancer Agents Med Chem (2015) 15:1269–76. 10.2174/1871520615666150518092840 25980814

[33] Wu JX Du WQ Wang XC Wei LL Huo FC Pan YJ. Rap2a Serves as a Potential Prognostic Indicator of Renal Cell Carcinoma and Promotes Its Migration and Invasion Through Up-Regulating P-Akt. Sci Rep (2017) 7:6623. 10.1038/s41598-017-06162-7 28747626 PMC5529368

