# Supplementary figures and images for: Long Non-Coding RNA LINC01569 Promotes Proliferation and Metastasis in Colorectal Cancer by miR-381-3p/RAP2A Axis
Source: Front Oncol. 2021 Aug 6;11:727698. doi: 10.3389/fonc.2021.727698 (PMC8378226; doi:10.3389/fonc.2021.727698)

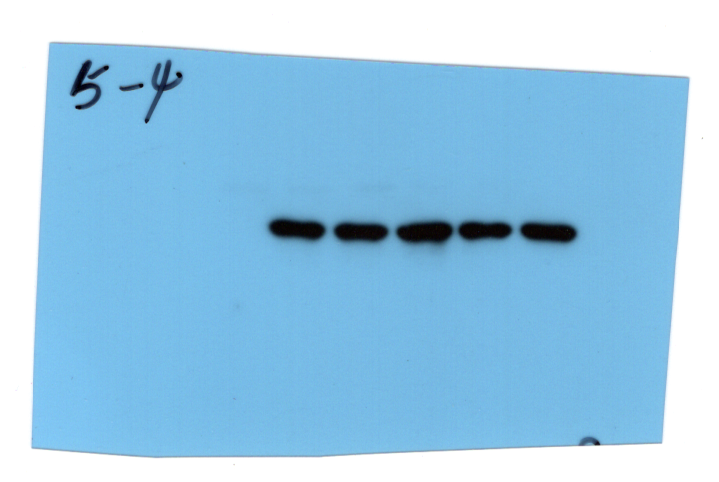

Supplement: Supplementary file 1 [file DataSheet_1.zip › Orignal Western blot/Figure 5/GAPDH-Figure 5C.tif]

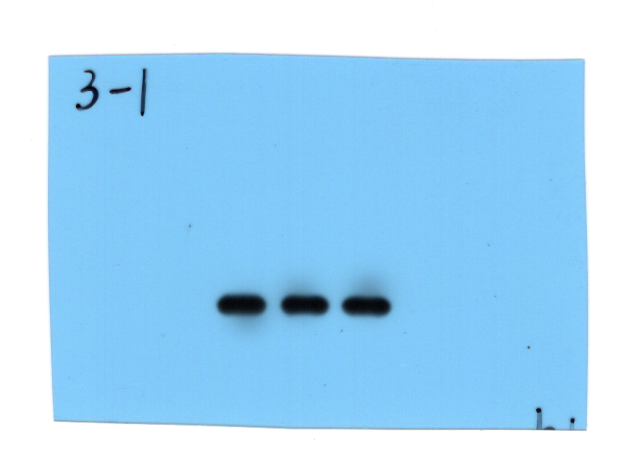

Supplement: Supplementary file 1 [file DataSheet_1.zip › Orignal Western blot/Figure 5/GAPDH-Figure 5E-hct116.tif]

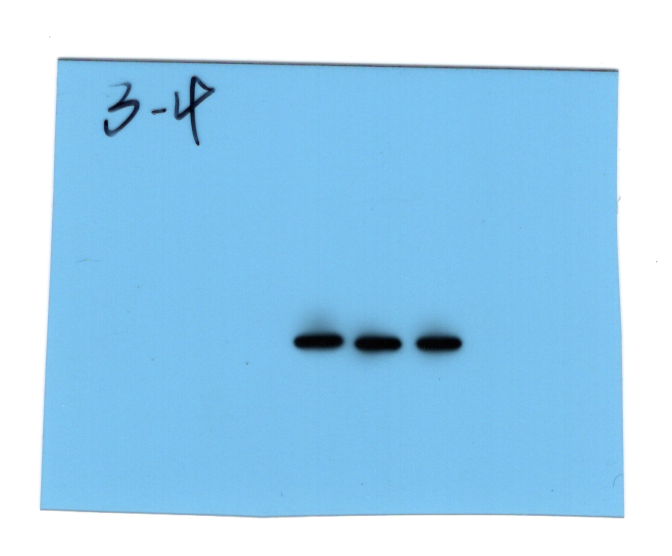

Supplement: Supplementary file 1 [file DataSheet_1.zip › Orignal Western blot/Figure 5/GAPDH-Figure 5E-sw620.tif]

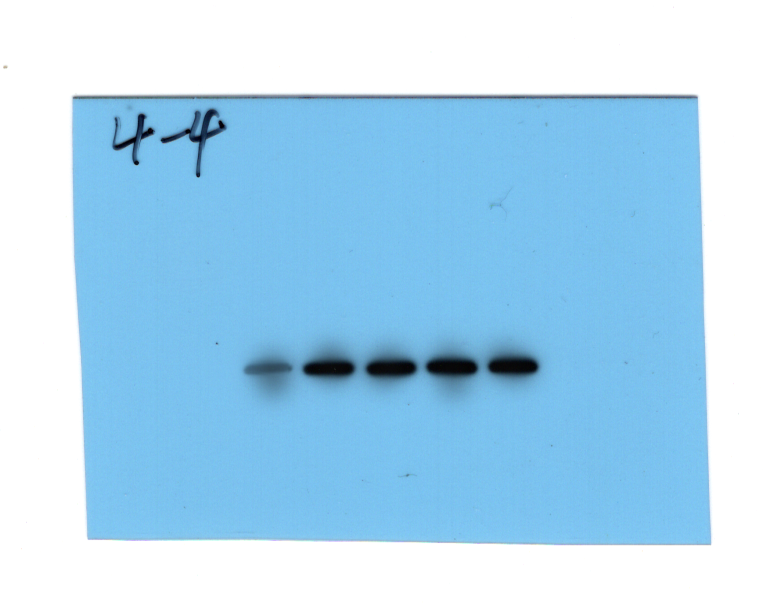

Supplement: Supplementary file 1 [file DataSheet_1.zip › Orignal Western blot/Figure 5/RAP2A-Figure 5C.tif]

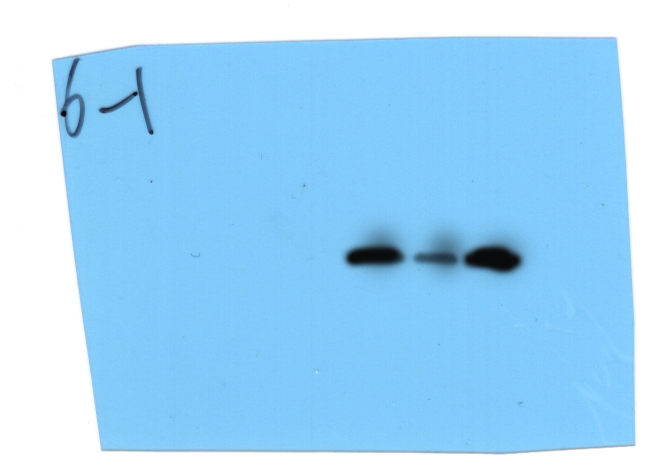

Supplement: Supplementary file 1 [file DataSheet_1.zip › Orignal Western blot/Figure 5/RAP2A-Figure 5E-SW620.tif]

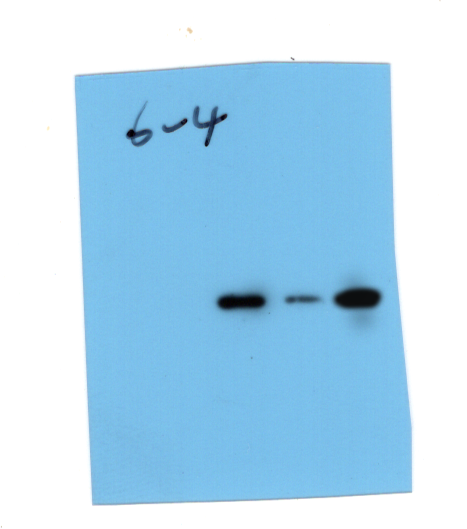

Supplement: Supplementary file 1 [file DataSheet_1.zip › Orignal Western blot/Figure 5/RAP2A-Figure 5E-hct116.tif]

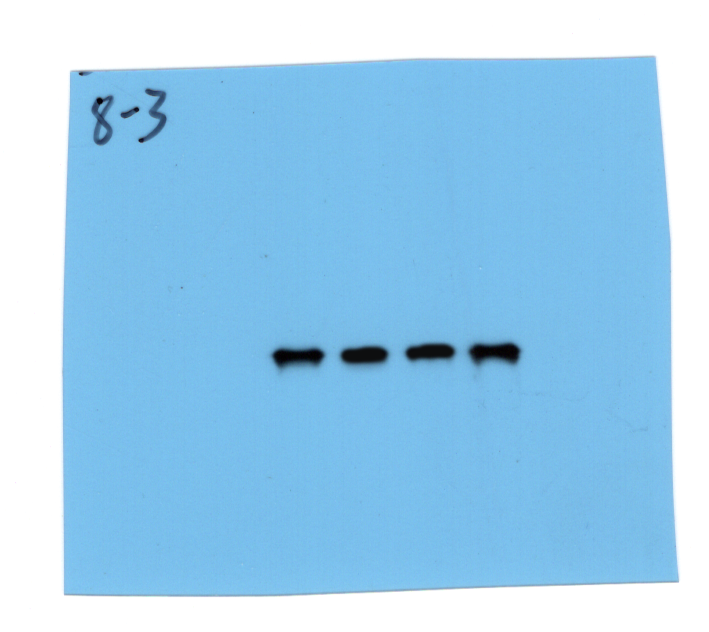

Supplement: Supplementary file 1 [file DataSheet_1.zip › Orignal Western blot/Figure 6/GAPDH-HCT116-Figure 6A.tif]

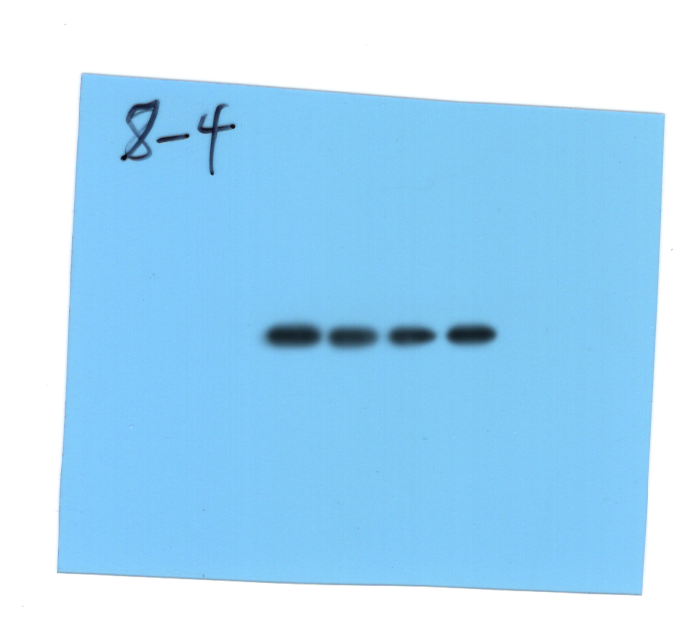

Supplement: Supplementary file 1 [file DataSheet_1.zip › Orignal Western blot/Figure 6/GAPDH-SW620-Figure 6A.tif]

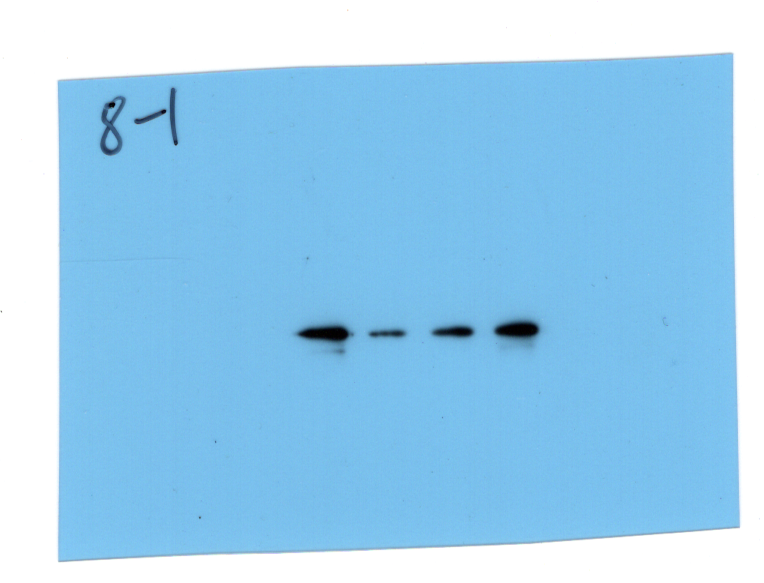

Supplement: Supplementary file 1 [file DataSheet_1.zip › Orignal Western blot/Figure 6/RAP2A-HCT116-Figure 6A.tif]

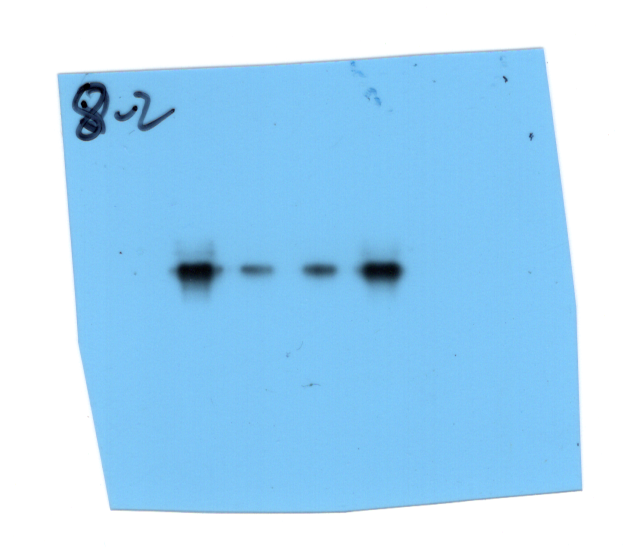

Supplement: Supplementary file 1 [file DataSheet_1.zip › Orignal Western blot/Figure 6/RAP2A-SW620-Figure 6A.tif]
